# Influences of Chemical and Nonchemical Stressors on Health and Quality of Life in Fenceline Communities: A Community-Based Participatory Research Survey in Southeastern Pennsylvania

**DOI:** 10.1089/env.2024.0078

**Published:** 2025-05-14

**Authors:** Andrea A. Chiger, Echo Alford, Kearni N. Warren, Eve S. Miari, Lora Snyder, Thom Nixon, Alexis Lightner, Ryan D. Kennedy, Mary A. Fox, Peter F. DeCarlo, Keeve E. Nachman, Sara N. Lupolt

**Affiliations:** Dr. Andrea A. Chiger is at the Department of Environmental Health and Engineering; also at the Department of Environmental Health and Engineering, Johns Hopkins Bloomberg School of Public Health, Baltimore, Maryland, USA. Mx. Echo Alford is at the Clean Air Council, Philadelphia, Pennsylvania, USA; also at Marcus Hook Area Neighbors for Public Health, Marcus Hook, Pennsylvania, USA. Ms. Kearni N. Warren is at the Clean Air Council, Philadelphia, Pennsylvania, USA; also at Marcus Hook Area Neighbors for Public Health, Marcus Hook, Pennsylvania, USA. Ms. Eve S. Miari is at the Clean Air Council, Philadelphia, Pennsylvania, USA; also at Marcus Hook Area Neighbors for Public Health, Marcus Hook, Pennsylvania, USA. Ms. Lora Snyder is at the Marcus Hook Area Neighbors for Public Health, Marcus Hook, Pennsylvania, USA. Mr. Thom Nixon is at the Marcus Hook Area Neighbors for Public Health, Marcus Hook, Pennsylvania, USA. Ms. Alexis Lightner is at the Department of International Health, Johns Hopkins Bloomberg School of Public Health, Baltimore, Maryland, USA. Dr. Ryan D. Kennedy is at the Department of Health Behavior and Society, Johns Hopkins Bloomberg School of Public Health, Baltimore, Maryland, USA. Dr. Mary A. Fox is at the Department of Environmental Health and Engineering; Risk Sciences and Public Policy Institute; also at the Department of Health Policy and Management, Johns Hopkins Bloomberg School of Public Health, Baltimore, Maryland, USA. Dr. Peter F. DeCarlo is at the Department of Environmental Health and Engineering, Johns Hopkins Whiting School of Engineering, Baltimore, Maryland, USA. Dr. Keeve E. Nachman is at the Department of Environmental Health and Engineering; Risk Sciences and Public Policy Institute; also at the Department of Health Policy and Management, Johns Hopkins Bloomberg School of Public Health, Baltimore, Maryland, USA. Dr. Sara N. Lupolt is at the Department of Environmental Health and Engineering; also at Risk Sciences and Public Policy Institute, Johns Hopkins Bloomberg School of Public Health, Baltimore, Maryland, USA.

**Keywords:** environmental justice, cumulative impact, community-based participatory research, fenceline community

## Abstract

**Background:**

Community organizers in Southern Delaware County, PA, expressed a desire to collect comprehensive data on environmental, health, and social conditions in their neighborhoods to inform advocacy efforts to prompt public health action.

**Methods:**

Using a community-based participatory research (CBPR) approach, our team of academic and community coinvestigators developed an online community health survey to characterize residents’ health concerns and the strengths, burdens, and needs of fenceline communities in Southern Delaware County. We included questions on chemical exposures, sources of pollution, financial stressors, health care, medical conditions, and priorities for policymakers.

**Results:**

Participants reported experiencing adverse effects of poor air quality, odors, and noise in their communities. Eighty-six percent of participants reported experiencing at least two nonchemical stressors, such as poor housing conditions, food insecurity, and experiences of racism and discrimination. We found high proportions of reported asthma diagnoses and symptoms in participants and the children living in their households. Symptoms of asthma, depression, and anxiety were more common than clinician diagnoses of these conditions. Participants also commonly reported decreased quality of life or functioning associated with physical and mental health issues.

**Discussion:**

Our findings highlight the importance of characterizing chemical and nonchemical stressors among residents in fenceline communities and expanding consideration of health to include acute symptoms, well-being, and quality of life. Our study was strengthened by our CBPR approach.

**Conclusion:**

Our work demonstrates the value of assessing cumulative impacts and employing CBPR approaches in fenceline communities.

## INTRODUCTION

In the United States, industrial sites such as chemical manufacturing plants and petrochemical infrastructure tend to be located nearby or adjacent to neighborhoods with high proportions of people of color and low-income residents.^1,2,3^ People living in these neighborhoods (hereafter: fenceline communities) often face numerous nonchemical stressors (e.g., poor housing conditions, food insecurity, and lack of access to health care) in addition to high levels of environmental pollution.^4,5,6^ A burgeoning body of literature demonstrates that these chemical and nonchemical stressors can act together on physiological systems to worsen health outcomes.^7,8,9^ For example, several studies have found that combined prenatal exposures to lead and psychosocial stress result in poorer neurodevelopmental outcomes in children compared with those exposed to either prenatal exposure alone.^10,11^ Moreover, proximity to pollution sources can be a significant source of stress, further decreasing the quality of life in fenceline communities.^12,13^

Considering cumulative impacts, defined as the “totality of exposures to combinations of chemical and nonchemical stressors and their effects on health, well-being, and quality of life outcomes,” is crucial to assessing the effects of pollution in fenceline communities.^14^ Historically, the practice of risk assessment in support of environmental decision making has been limited to consideration of chemical exposures, with little regard for nonchemical stressors that can increase population susceptibility and vulnerability.^15^ The recent national focus on systemic racism and increased recognition of the need for environmental justice^16,17^ has prompted renewed efforts by governmental agencies at the state and federal levels to shift toward cumulative impact assessment.^18,19^

Cumulative impact assessment broadens recommended approaches and data sources in support of environmental decision making to include community-based participatory research (CBPR),^20^ defined as a collaborative approach to research that aims to engage communities in all aspects of the research process.^21,22^ This recommendation acknowledges that external researchers may fail to understand the complexities of real-world exposures and impacts; CBPR is key to providing the local knowledge and insights needed to support cumulative impact assessment.^23^ A key principle of CBPR is that research should be conducted to influence policy actions, social change, or related outcomes and to benefit all members of the partnership.^24^ Active participation in the research process can empower community members and build community knowledge, skills, and capacity for additional research opportunities; learning occurs on both sides of the community–academic partnership.^25^

**FIG. 1.**
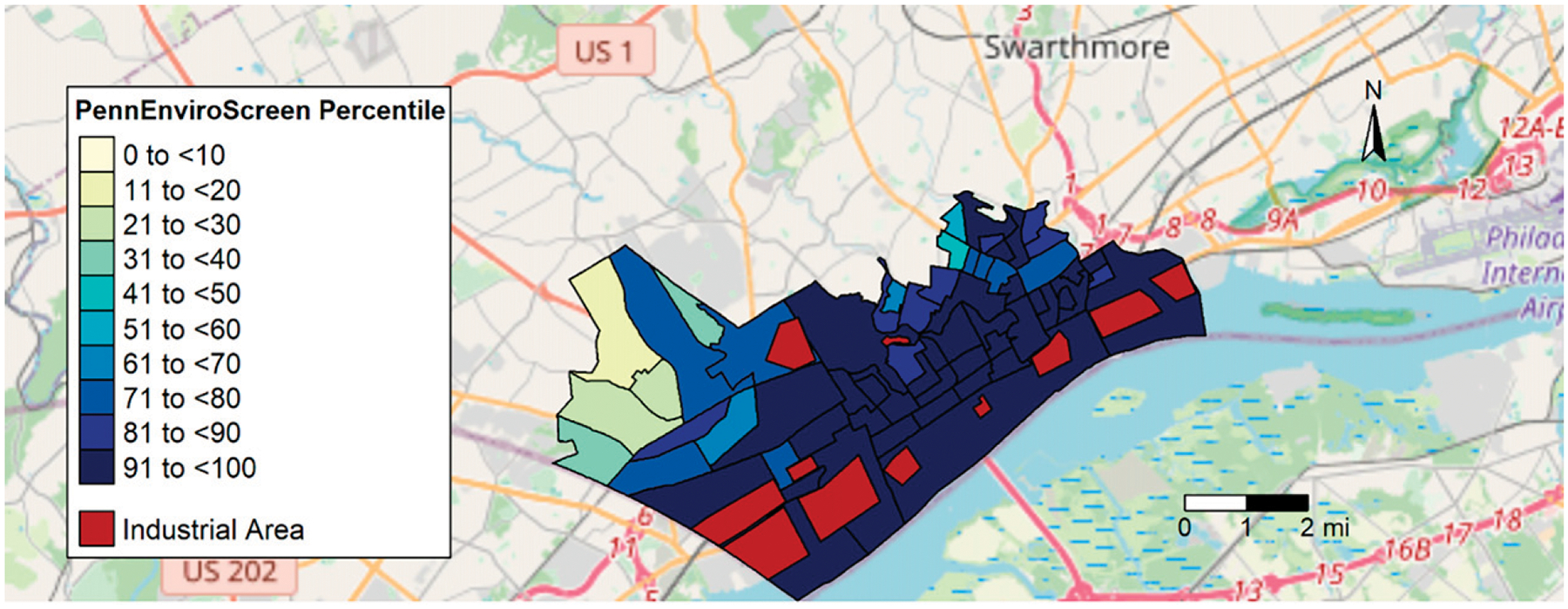
Map of study area showing industrial facilities and PennEnviroScreen scores (environmental justice areas are defined as block groups at or above the 80^th^ percentile).

Fenceline communities in Southern Delaware County, Pennsylvania, (hereafter: SoDelCo) have endured elevated pollution levels for decades.^26,27^ Although our study area is limited to approximately 20 square miles, it is home to numerous industrial facilities (Fig. 1), including chemical manufacturing plants, petrochemical infrastructure, and two large incinerators.^28,29^ ReWorld^™^ Delaware Valley, a trash incinerator located in the City of Chester, PA, emits greater amounts of PM_2.5_ than any other such facility in the nation^30^ and has a long history of violations and noncompliance with environmental regulations.^31^ The expansion of industrial facilities in SoDelCo^32,33^ has occurred, even though these fenceline communities are classified as environmental justice areas by the Pennsylvania Department of Environmental Protection’s Environmental Justice Mapping and Screening Tool (PennEnviroScreen) (Fig. 1).^34^ Residents face numerous nonchemical stressors that can increase vulnerability to chemical exposures. SoDelCo communities have high proportions of residents who live below twice the federal poverty level, lack health insurance, experience housing burdens (i.e., pay over 50% of their income on housing costs), and have low educational attainment.^35^ The co-occurrence of numerous chemical and nonchemical stressors in SoDelCo underscores the need for holistic consideration of influences on health, quality of life, and well-being.

Our objective was to characterize cumulative impacts in SoDelCo by developing and implementing a community and environmental health survey using a CBPR approach. Specifically, we aimed to characterize chemical stressors, nonchemical stressors, and health, well-being, and quality of life outcomes. We also aimed to explore racial disparities in exposures and linkages between stressors and health.

## METHODS

### CBPR approach

In August 2021, Johns Hopkins University (JHU) researchers began collaborating with community organizations in SoDelCo on community-engaged air monitoring studies and educational campaigns on understanding and reporting pollution.^36^ The present partnership was built upon these existing relationships. As community partners expressed interest in conducting surveys and focus groups, we expanded our JHU team to add expertise in these areas. JHU researchers completed readings recommended by community partners^37,38^ and learned about community context. Together, our team refined our research objectives and successfully applied for a pilot grant, which included dedicated funding for community investigators and organizations.

To foster our partnership and build trust, JHU investigators attended local events and engaged in regular meetings held by established community organizations in SoDelCo. Although community investigators took the lead on specific steps of the research process (e.g., outreach and recruitment) and academic investigators on others (e.g., data analysis), all investigators were given equal opportunities to participate in all aspects. Moreover, investigators from both sides of the community–academic partnership participated in every step. Our team met weekly during the survey study period and periodically evaluated the strengths and limitations of our approach, partnership, and outputs.

### Survey design and implementation

Adults over 18 years old who resided in select SoDelCo municipalities were eligible to participate. The list of eligible municipalities was developed by community investigators and based on perceptions of proximity to polluting sources. Our survey was designed to capture information on participant demographics, pollution and other environmental exposures, other individual- and community-level stressors, and health diagnoses and symptoms of participants and the children living in their households. It consisted of multiple-choice, checklist, Likert scale, and open-ended questions and was designed to be completed in 20–25 minutes. We obtained survey questions from previously validated sources^39,40,41,42,43,44,45,46,47,48,49,50,51,52^ and developed questions to address specific community concerns and local practices as needed (Supplementary Data). For example, to investigate concerns about odors in the ambient air, we asked participants to rate the following on a 5-point Likert scale ranging from “never” to “always”: (1) how often they notice odors in their community, (2) how often odors cause them physical discomfort or symptoms, and (3) how often odors impact their mental well-being. We also included an open-ended question for participants to qualitatively describe the odors.

Our survey was implemented online using Research Electronic Data Capture (REDCap), a secure method for data collection with a user-friendly web interface.^53,54^ To minimize missing data, all questions were programmed as required in REDCap. For multiple-choice questions about potentially sensitive topics (e.g., food insecurity), a “prefer not to say” option was added. Survey participants were compensated with a $10 gift card and invited to enter a raffle to win an additional $50. The Johns Hopkins Institutional Review Board (IRB No. 24902) reviewed and approved all study tools and protocols.

We recruited survey participants between June 2023 and February 2024. To minimize self-selection and reporting bias, recruitment efforts for our survey (entitled “Community and Environmental Health Survey”) did not provide details of its contents. Our initial recruitment strategy consisted primarily of social media posts and email lists of our community partner organizations. However, we revised our strategy after our survey was inundated by hundreds of bot responses in rapid succession within a few hours of posting the survey link to a public social media account. To prevent further bot responses, we enlisted a contractor to lead in-person outreach at various community events (e.g., festivals and local council meetings) and to leverage personal networks. In addition, we modified our eligibility screener to require a password as a condition for advancing to the survey. We created distinct passwords for each recruitment activity, enabling recruitment to continue even if a password was compromised. During all outreach efforts, we emphasized to potential participants that the link and password should not be shared on social media.

To avoid including bot responses in our data analysis, we manually checked each response and excluded those with irregularities in timing, internal inconsistency (e.g., large misalignment between age and year of birth), or inappropriate responses to open-ended questions.

### Data analysis

Statistical analyses were conducted using R version 4.3.1. We calculated frequencies for categorical variables and descriptive statistics for numerical variables. To investigate racial/ethnic disparities, we compared distributions of chemical and nonchemical stressors by race/ethnicity (as a proxy for structural racism and discrimination) using one-sided Mann–Whitney *U* tests for Likert scale data and two-sample proportion tests for binary variables. Due to the racial/ethnic composition of our study area^55^ and our small sample size, these analyses were limited to Black and White participants. We conducted ordinal regression to assess the relationship between frequency of odor detection and length of residence, controlling for age and gender, to investigate whether residents may become habituated to odors in their communities.

We conducted logistic regression models to investigate associations between cumulative exposures to stressors and health, controlling for age, gender, smoking status, and length of residence in SoDelCo. To characterize cumulative exposures, we summed the total number of nonchemical stressors faced by each participant (housing quality issues, noise disruptions, food insecurity, racism or discrimination, financial insecurity, lack of transportation, violent crime, and lack of health insurance) and categorized participants as having low, medium, or high cumulative exposures. All participants were assumed to be exposed to chemical stressors. Our dependent variables were presence of any physical health symptoms within the past 30 days and diagnosis of any medical conditions while living in SoDelCo.

Responses to open-ended survey questions were analyzed using an inductive coding approach in Dedoose version 9.0.17. We reviewed each response and identified emergent themes and then coded each response using an iterative process.

## RESULTS

Table 1 displays demographics of the 143 participants who completed the survey. The median age of participants was 42 years, slightly above the median of 38 years among overall residents of our study area.^56^ Participants resided in SoDelCo for a mean of 30 years (standard deviation = 19 years). Seventy-eight percent of our study participants identified as female. Most participants reported no Hispanic ethnicity (92%) and identified as White (62%), which is similar to the overall racial and ethnic distribution of residents living in our study area (94% non-Hispanic, 62% White, and 27% Black). Half of participants reported having at least one child under 18 living in their household.

### Exposure to chemical stressors

Most participants (88%) reported feeling very concerned about pollution in their communities. Moreover, 51% of participants indicated that pollution in their communities was among the top three factors most negatively impacting their health and 45% believed it should be among the top three issues for policymakers to prioritize. General perceptions of poor air quality were associated with physical and mental health symptoms, respectively, in 69% and 45% of participants, with Black participants significantly more likely to report physical symptoms than White participants (W = 2399, *p* < 0.01).

Eighty-five percent of participants indicated that they were regularly (i.e., sometimes, often, or always) able to detect odors in the ambient air in their communities. Open-ended descriptions of these odors included descriptors of rotten eggs (18%), gas (16%), burning (16%), chemicals (11%), trash (10%), and sewage (8%). Three-quarters of participants reported that odors were regularly associated with physical discomfort or symptoms, whereas 50% reported decreased mental well-being. Black participants reported more frequent exposures to odors (W = 2460, *p* < 0.01) and subsequent adverse physical (W = 2581, *p* < 0.01) and mental health (W = 2199, *p* < 0.01) symptoms than White participants. No significant differences were observed in odor frequency by length of residence in SoDelCo.

### Exposure to nonchemical stressors

Approximately half of participants reported regular disruption of daytime activities (53%) or sleep (48%) due to environmental noise. This disruption was regularly associated with physical symptoms in 56% of participants and with decreased mental well-being in 41%. Comparing results by race, Black participants were more likely than White participants to experience disruption of daytime activities (W = 2287, *p* < 0.01) and sleep (W = 2195, *p* < 0.01) and associated physical health symptoms (W = 2271, *p* < 0.01).

**FIG. 2.**
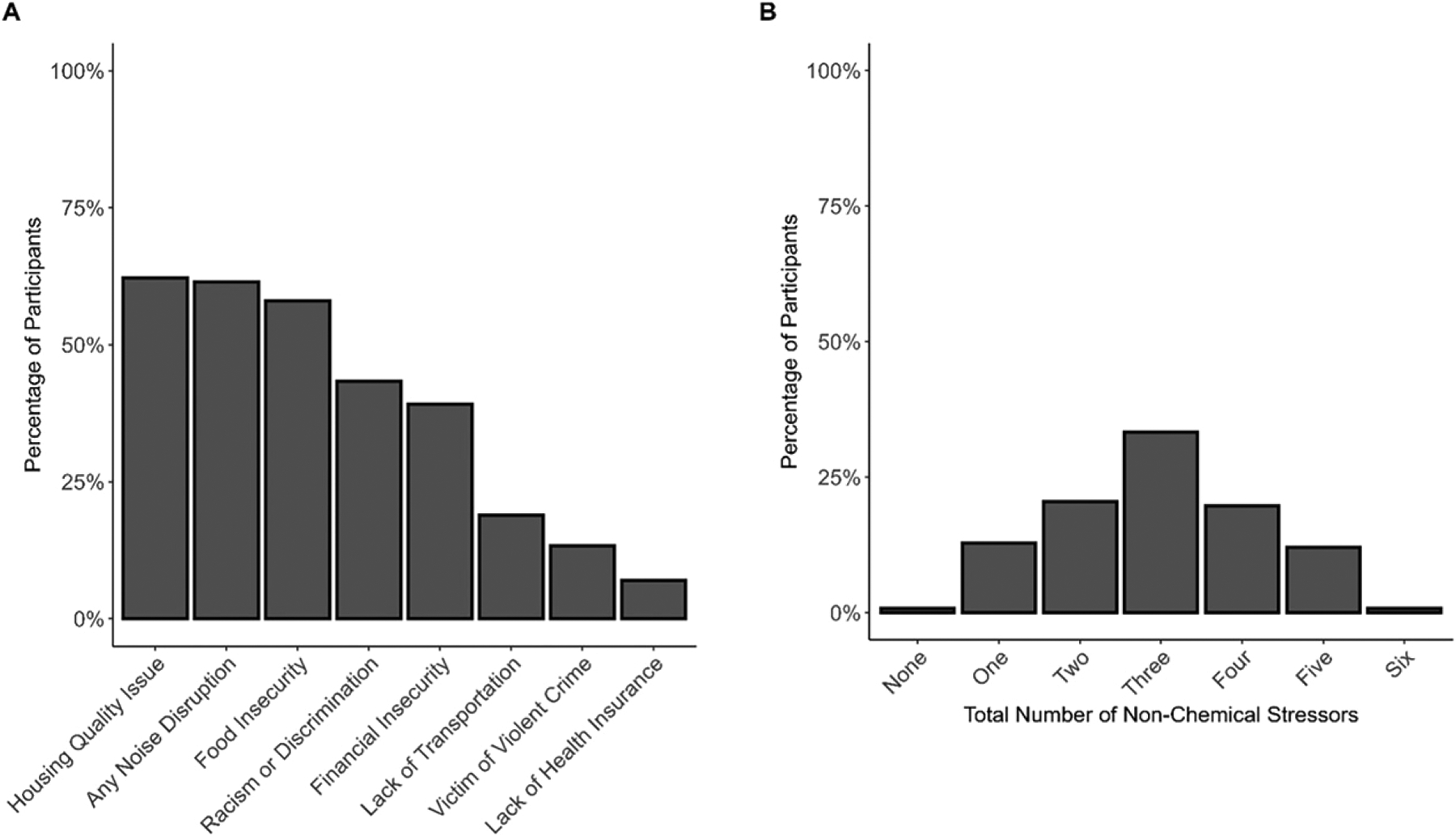
Percentage of participants who reported various types of nonchemical stressors **(A)** and the number of total nonchemical stressors reported per participant **(B)**.

Participants also reported exposure to numerous stressors associated with social and economic inequities. Over half of participants reported issues with housing quality (62%) or food insecurity (58%), and nearly half reported experiences of racism or discrimination (43%) and financial insecurity (39%; Fig. 2). Moreover, most participants (86%) reported current exposure to more than one stressor (Fig. 2). Of these stressors, participants identified limited financial resources (29%) and violence and/or crime (27%) as having the greatest negative impact on their health. Twenty-nine percent of participants had “medium low” financial well-being, indicating minimal savings, difficulty making ends meet, and issues with obtaining credit. Thirteen percent of participants identified as victims of violent crime while living in SoDelCo. Black participants were significantly more likely to be violent crime victims than White participants (W = 2807, *p* < 0.01).

Nearly two-thirds of participants (62%) reported one or more issues with their current housing situation, most commonly the need for minor or major repairs (28% and 22%, respectively), bugs or rodents (24%), or mold (18%). Black participants were more likely to report issues with housing than White participants, although no significant differences between groups were observed except for mold (Z = 3.54, *p* < 0.01). Of the 56 renters in our sample, 9% reported being at risk of being evicted from their rental properties. Three percent of participants did not have a steady place to live.

Participants reported worrying about running out of food and/or being unable to buy sufficient food, indicating that 58% were at risk for food insecurity (Fig. 2). Around 10% of participants did not have consistent access to reliable transportation in the past 12 months, which kept them from medical appointments or filling medications (9%) or from work, nonmedical appointments, or obtaining other needed items (13%). Most participants had health insurance (93%), and few (7%) reported being denied insurance coverage in the past 3 years. However, participants reported waiting 3 or more months for a medical appointment (29%) or refusing recommended medical treatment or medications because of the cost (21%) within the past 3 years. One-quarter of participants reported they could not complete the desired education or career skills training because of costs or other barriers.

### Medical diagnoses and health symptoms

**FIG. 3.**
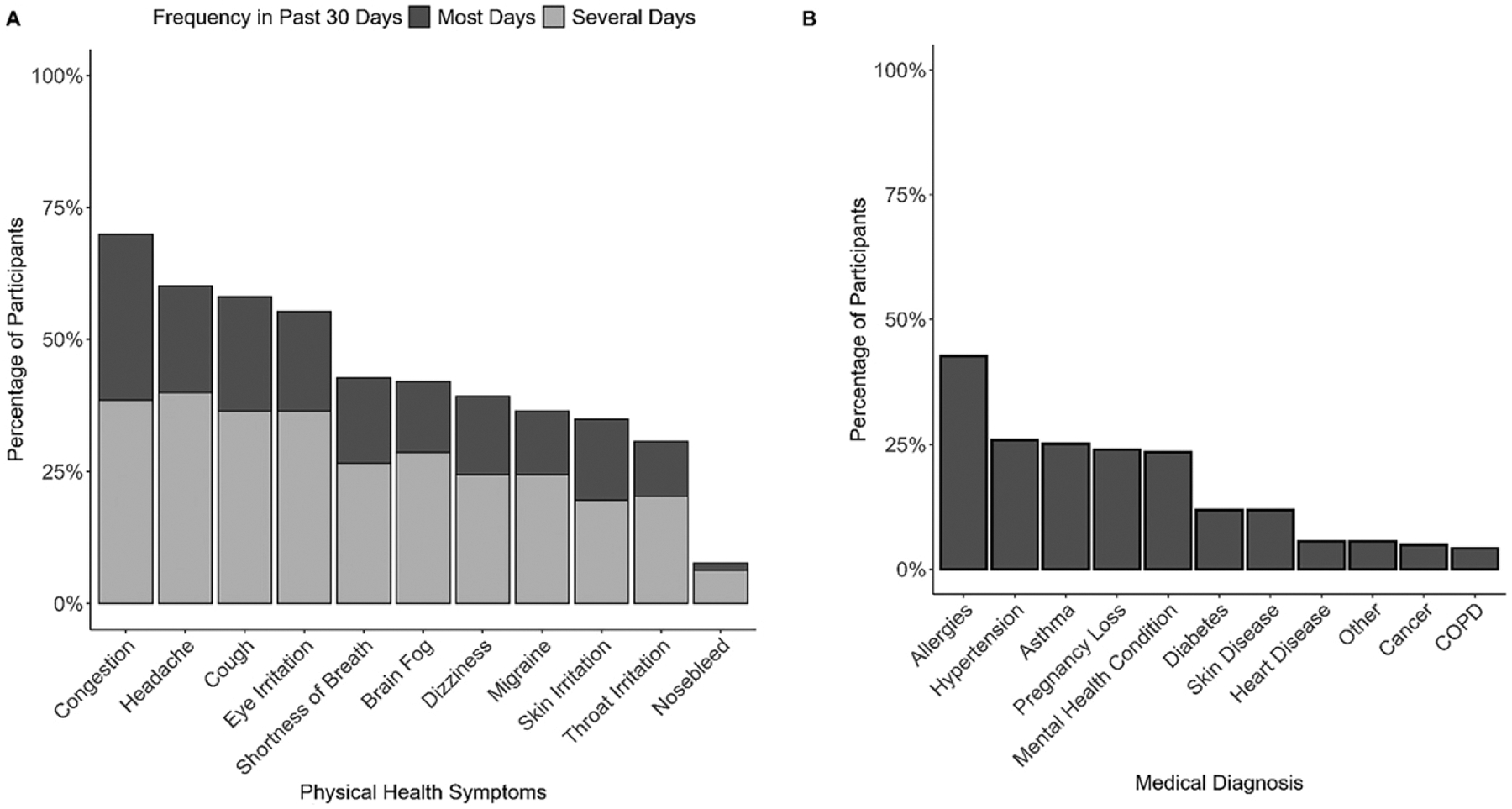
Percentage of survey participants who reported various physical health symptoms several or most days within the past month **(A)** and medical diagnoses of health conditions while residing in Southern Delaware County **(B)**.

The most common medical diagnoses received by participants while living in SoDelCo were allergies (43%), hypertension (26%), asthma (25%), and mental health conditions (24%; Fig. 3). Among participants identifying as female, 23% had suffered one or more pregnancy losses, including one or more miscarriages and/or stillbirths. Few participants reported cancer (5%) or heart disease (6%). Although only 6% of participants reported physician-diagnosed long COVID, 16% believed that they were experiencing effects from long COVID, and an additional 13% were unsure.

Participants regularly reported adverse physical health symptoms: except for nosebleeds, each of the self-reported health symptoms included in our survey was experienced by at least 30% of participants within the past month. The three most reported health symptoms were congestion (70%), headaches (60%), and cough (58%). Moreover, around 10% of participants experienced one or more of these three symptoms every day. Physical health issues were associated with impaired daily functioning among participants. Half of participants reported feeling like they accomplished less than they would like due to their physical health, and one-third reported being limited in the types of work or activities they could engage in or having difficulty performing them. Nonetheless, only 28% classified their health as fair or poor instead of good to excellent.

Participants’ self-reported symptoms of depression and anxiety suggest that these issues might be more common in our sample than what is reflected by medical diagnoses. Many participants reported feelings consistent with depression (37%), anxiety (50%), or both (54%). Symptoms of depression and anxiety were classified as mild in 31% of participants, moderate in 15%, and severe in 8%. Forty-five percent of participants felt that their mental health prevented them from accomplishing as much as they could like. In addition, one-quarter of participants cut down on the amount of their work or did not conduct work or other activities as carefully as usual. Most participants (60%) felt that their mental and physical health issues interfered with their normal social activities; one-quarter of participants indicated that this impairment was moderate or severe.

**FIG. 4.**
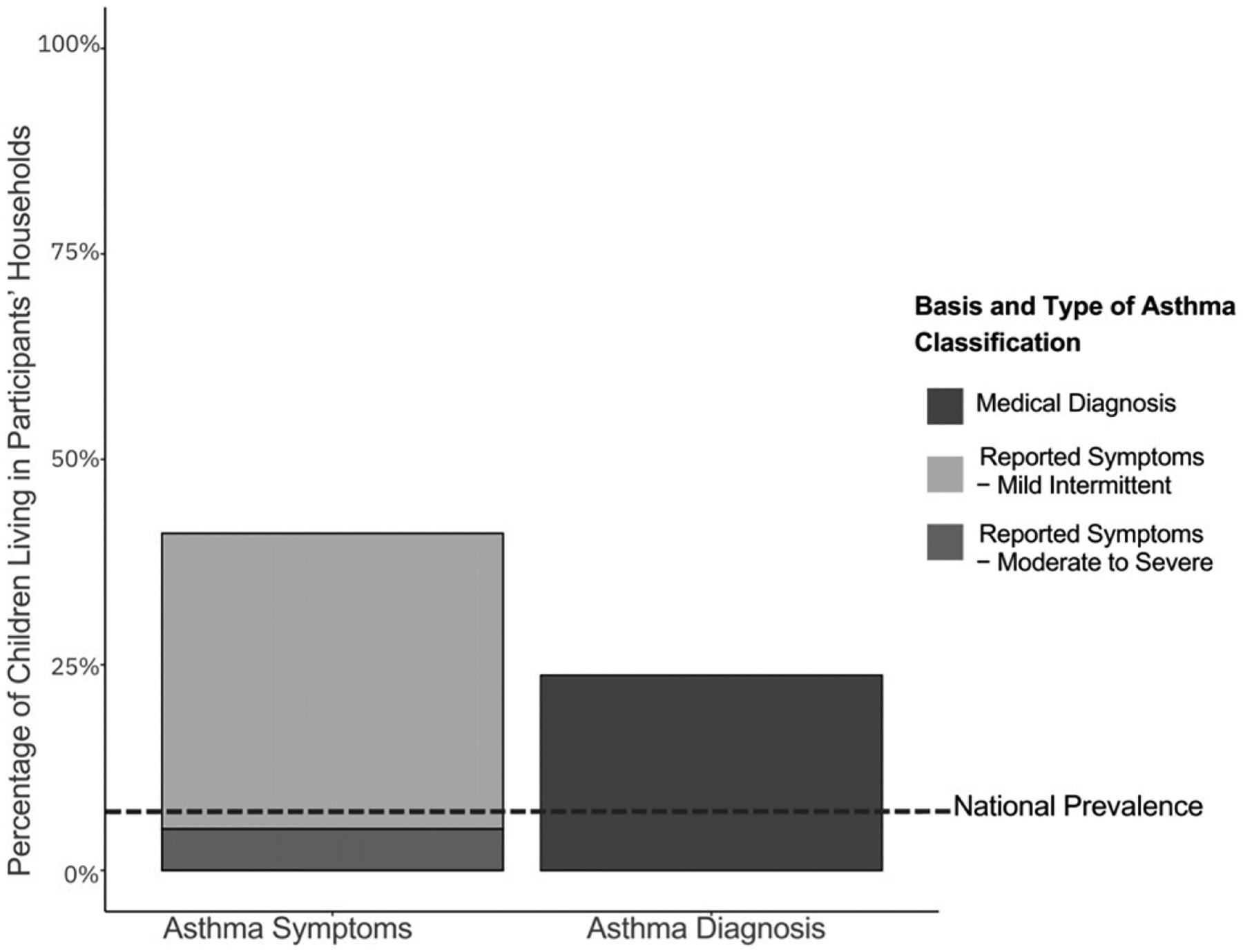
Percentage of children living in the households of participants who have asthma, based on reported symptoms classified using the National Asthma Education and Prevention Program methodology or on medical diagnoses and compared with the national average prevalence of asthma in children.

Among children residing with participants (*n* = 139), 24% received an asthma diagnosis while living in SoDelCo (Fig. 4). However, when we rated reported respiratory symptoms per National Asthma Education and Prevention Program guidelines,^57,58^ we found that 38% of children met criteria for mild intermittent asthma and 8% for moderate to severe asthma. Nine percent of children were diagnosed with anxiety or depression. Autism spectrum disorder emerged as an additional concern, with nine reported cases (6%). Children were reported to have a high prevalence of physical health symptoms during the past month, most commonly headaches (49%) and congestion (47%). Eye, skin, and throat irritation were each reported for 25%–30% of children. Fourteen percent of children were reported to have experienced nosebleeds during the past month.

### Cumulative impacts

Two-thirds of participants reported experiencing three or more of the seven nonchemical stressors assessed in our study (Fig. 2). After controlling for age, gender, smoking status, and length of residence in SoDelCo, we found significant associations between increases in cumulative exposures and presence of clinician-diagnosed medical conditions (odds ratio [OR] = 1.83, 95% confidence interval [CI] = 1.04, 3.33). We also observed a trend between increased cumulative exposures and presence of adverse physical health symptoms within the past 30 days, although the association failed to reach significance (OR = 1.40, 95% CI = 0.89, 2.22).

## DISCUSSION

Using a CBPR approach, our study provides insight into the numerous chemical and nonchemical stressors in SoDelCo that can decrease health, well-being, and quality of life. Noxious odors, which were both frequently observed and associated with physical and mental harm, emerged as a primary environmental health concern among participants. Our results indicate that residents did not habituate to odors in their communities over time. We also found evidence of racial disparities in exposures to odors and subsequent effects. Although noxious ambient odors have also been found in other studies of fenceline communities,^59,60,61^ they remain understudied as exposures in environmental epidemiological studies^62^ and ignored in the traditional risk assessment framework. Our results highlight the need to expand consideration of chemical exposures beyond their measured concentrations to include assessments of associated odors. Moreover, our investigation of cumulative exposures to nonchemical stressors showed that participants who faced a greater number of stressors had poorer health outcomes. Our findings underscore the need for a paradigm shift toward cumulative impact assessment, which considers exposures in a holistic manner.

Our work also provides support for another key aspect of cumulative impact assessment: expanding consideration of health beyond disease and chronic physical effects to include acute physical and mental health symptoms, well-being, and quality of life. Nosebleeds occurred in 8% of our sample during the past month and have been highlighted as a concern in SoDelCo and other communities on the fenceline of petrochemical facilities.^63,64^ Between 30% and 70% of participants experienced health symptoms such as congestion, headaches, and feelings of depression and anxiety several or more days during the past month. Moreover, we found that physical and mental health issues commonly interfered with the ability of participants to accomplish their work and engage in social activities. However, none of these impacts would be captured in a traditional risk assessment framework in support of environmental decision making. A narrow focus on disease and chronic physical health effects obscures the true burden of stressors on residents of fenceline communities.

Our findings on asthma align with community concerns and prior research^65,66^ and indicate that actions to address this issue are needed. We found a high prevalence of asthma diagnoses among both adults (25%) and children (24%) in our sample, which is similar to a 2018 study that found an asthma prevalence of 26.8% in children living in one municipality of our study area. Our estimates are 3.1 and 3.7 times higher than the average asthma prevalence of U.S. adults and children, respectively.^67^ Additionally, we found that 46% of children experienced symptoms consistent with asthma over the past year. Over one-fifth of participants reported that their homes were infested with rodents or cockroaches and had mold issues, contributing to and exacerbating respiratory issues.^68^ Considered in the context of environmental pollution and asthma rates, this finding underlines the double jeopardy of coexposures to multiple stressors in fenceline communities and their contribution to health disparities.

Our study has several limitations, including the small sample size and potential for selection and recall bias. Our use of convenience sampling limits the representativeness and generalizability of our study and introduces the potential for selection bias. As certain population groups (e.g., women, residents of specific municipalities) were oversampled, our results lack representativeness for all populations within our study area. Recruitment via email lists of community investigator organizations may also have introduced selection bias, as these participants may have been more concerned about environmental issues than a typical resident; however, these responses represented only a fraction (7%) of our final sample. Over half of respondents (55%) were recruited via in-person or social media outreach led by a contractor who was not previously involved in environmental or public health advocacy. We relied on self-reported data for exposures and outcomes, which may have resulted in over- or underestimates of true values, although concordance between self-reported health data and medical records tends to be high.^69,70^ However, it should be noted that our estimates and findings align with previous investigations in SoDelCo.^71,72^ Specifically, the Delaware County Health Department’s 2023 Community Health Assessment also identified concerns about mental health and access to mental health services as key concerns. While the County’s assessment was focused on a broader range of health issues, an important difference was our survey’s focus on environmental health concerns. Our small sample size further limits generalizability and reduces our statistical power. According to anecdotal feedback from residents, the length of our survey was a barrier to participation. Future studies should explore how to better balance potential burden on participants with the time required to capture information on cumulative impacts.

Our issues with bot responses highlight the difficulties of implementing online surveys in an age of rapid advancements in artificial intelligence. Common methods for preventing bots reported in the literature^73^ (e.g., captchas and basic knowledge questions) were ineffective for us. Instead, we recommend researchers require respondents to provide a password to advance to the survey. Different passwords should be used for each recruitment event or activity; if one password is compromised, it can easily be removed from the survey code with minimal disruption to recruitment. We also recommend setting up email alerts for survey completion, as bot responses are typically completed in rapid succession. To avoid erroneous inclusion of bot responses, researchers should manually check each survey response for timing irregularities, internal inconsistency, and inappropriate responses to open-ended questions. While we believe that these strategies were successful in preventing and excluding bot data, we acknowledge the potential for bot responses as an additional limitation of our study.

A key strength of our study was our CBPR approach, which increased the relevance and reach of our work.^74^ It allowed us to effectively develop and tailor survey design and implementation to community needs, priorities, and preferences, enhancing the relevancy of our study for SoDelCo residents. Prior to publication of this article, the main findings were shared with key stakeholders through a technical report and community meeting. The 60-page technical report is publicly available and contains over 30 graphics to make our survey findings more engaging, accessible, and understandable to the broader community.^75^ Additionally, it includes recommendations for state and local regulators and policymakers, community organizations and advocates, and academic researchers. These efforts have expanded the reach of our study beyond traditional academic audiences, increasing the likelihood of future advocacy and action to address cumulative impacts.

Reflecting upon our community–academic partnership, we developed recommendations for future researchers engaging in CBPR in fenceline communities. It is important to establish a team with diverse backgrounds, skills, and racial and gender identities; throughout our conversations, the diversity of our team was recognized as foundational to our success. In addition, academic investigators must be willing to spend time and effort to attend community events and build relationships with community investigators. Our efforts to consistently attend community meetings and other events helped foster trust. Together, we learned more about our study area’s complex history and the interpersonal contexts of environmental justice efforts to date, which directly informed our study design. Academic investigators must be mindful that communities are not a monolith and that building coalitions is a process. Our team’s frequent and flexible communication helped ensure that each investigator had ample opportunities to provide feedback. Academic coinvestigators should be cognizant of the fact that mobile messaging options are often more convenient for community investigators with roots in community organizing. We also recommend using the title of “investigator” rather than “partner” for team members on both sides of the community–academic partnership; this language better reflects and recognizes meaningful involvement in the scientific process.

## CONCLUSION

Our findings shed light on chemical (e.g., air pollution and odors) and nonchemical stressors (e.g., noise, housing quality, and food insecurity) in SoDelCo. In addition, our work demonstrates that a focus on physical effects and disease fails to adequately characterize the health, well-being, and quality of life issues in fenceline communities. Further, we found evidence that participants facing a greater number of nonchemical stressors reported more symptoms and diagnoses than those facing fewer nonchemical stressors. Our work underscores the need for a shift to cumulative impact assessment in environmental decision making and an expansion of CBPR approaches in academic studies.

## Supplementary Material

Supplementary Material

Supplementary Data

## Figures and Tables

**Table 1.** Demographic Characteristics of ASSESS Participants (*n* = 143)

*Characteristic*	n *(%)*
Gender identity	
Female	111 (78)
Male	30 (21)
Nonbinary/gender nonconforming	1 (1)
Prefer not to say	1 (1)
Transgender	
No	138 (97)
Yes	3 (2)
Prefer not to say	2 (1)
Age (years)	
18–29	23 (16)
30–39	36 (25)
40–49	32 (22)
50–59	20 (14)
60 and above	32 (22)
Race	
White	89 (62)
Black or African American	39 (27)
Multiracial	7 (5)
American Indian or Alaskan Native	2 (1)
Native Hawaiian	1 (1)
Some other race	5 (3)
Hispanic ethnicity	
No, not of Hispanic, Latino, or Spanish origin	131 (92)
Yes, Mexican, Mexican American, or Chicano	2 (1)
Yes, Puerto Rican	5 (3)
Yes, another Hispanic, Latino, or Spanish origin	4 (3)
Yes, Cuban	1 (1)
Municipality of residence	
Trainer	52 (36)
Marcus Hook	28 (20)
City of Chester	25 (17)
Linwood [Lower Chichester]	17 (12)
Upper Chichester	9 (6)
Boothwyn	5 (4)
Chester Township	2 (1)
Eddystone	2 (1)
Parkside	1 (1)
Twin Oaks	1 (1)
Upland	1 (1)
Residence length (years)	
0–14	36 (25)
15–29	31 (22)
30–44	46 (32)
45 and above	30 (21)
