# Supplementary material for: Influences of Chemical and Nonchemical Stressors on Health and Quality of Life in Fenceline Communities: A Community-Based Participatory Research Survey in Southeastern Pennsylvania
Source: Environ Justice. Author manuscript; Available in PMC 2025 Aug 28. (PMC12381651; doi:10.1089/env.2024.0078)
Supplement: Supplementary Material [file NIHMS2088118-supplement-Supplementary_Material.docx]

**Survey Questions**

**Basic Information**

1. What are the cross-streets nearby to your home? Please provide both street names.

____________________________________

(Note: this information will be securely stored and only visible to limited Johns Hopkins University researchers.)

1. How many years have you lived in Southern Delaware County?

______________

1. What is your age (in years)?

______________ (Please enter a whole number.)

1. What is your gender identity?

Woman

Man

Non-Binary/Gender Non-Conforming

Other

Prefer not to say

1. Are you transgender?

Yes

No

Prefer not to say

1. Are you of Hispanic, Latino, or Spanish origin?^[[1]](#footnote-1)^

No, not of Hispanic, Latino, or Spanish origin

Yes, Mexican, Mexican American, or Chicano

Yes, Puerto Rican

Yes, Cuban

Yes, another Hispanic, Latino, or Spanish origin

1. What is your race? Please select all that apply.^[[2]](#footnote-2)^

White

Black or African American

American Indian or Alaskan native

Chinese

Vietnamese

Native Hawaiian

Filipino

Korean

Samoan

Asian Indian

Japanese

Chamorro

Other Asian

Some other race

1. Have you smoked at least 100 cigarettes or vaped at least 100 times in your entire life?^[[3]](#footnote-3)^

Yes

No

Unsure

Prefer not to say

1. Do you now smoke cigarettes or vape?^[[4]](#footnote-4)^

Every day

Some days

Not at all

Prefer not to say

1. Including yourself, how many people in your household smoke cigarettes, vape, or use any other tobacco product? (Please enter a whole number. If you prefer not to answer, please enter 999.)

_________________________

1. Including yourself, how many people in your household smoke cigarettes, vape, or use any other tobacco product inside the house? (Please enter a whole number. If you prefer not to answer, please enter 999.)

_________________________

**Pollution and Other Environmental Exposures**

1. In three words or less, what comes to mind when you think about the environment in your municipality?

__________________________________

2. How concerned are you about pollution/harmful chemicals in your municipality? [One choice]

Not concerned

Somewhat concerned

Very concerned

Unsure

Not applicable; I was unaware of pollution/harmful chemicals in my community.

2. a) Where do you think the pollution in your community comes from? For example, what specific facilities or other sources are contributing to the pollution?^[[5]](#footnote-5)^

________________________

2. b) What first brought your attention to the pollution/harmful chemicals in your municipality? ^[[6]](#footnote-6)^

Drinking water taste or smell

Health symptoms

Odors in the air

Reports in the news

Talking to others in your community

Unsure

Other

2. c) How do you think you’re being exposed to pollution/harmful chemicals in your community? Please select all that apply. If other, please specify.^[[7]](#footnote-7)^

Drinking water

Dust

Food supply

Indoor air

Outdoor air

Soil

Other

3. How often do you notice odors in your municipality?

Always

Often

Sometimes

Rarely

Never

3. a) Please describe the odors you notice in your municipality.

4. How often do noises in your municipality affect your daytime activities?

Always

Often

Sometimes

Rarely

Never

5. How often do noises in your municipality affect your sleep?

Always

Often

Sometimes

Rarely

Never

6. How often have the following issues caused you physical discomfort or symptoms?

Always - Often - Sometimes - Rarely - Never

6.1 Noise

6.2 Odors

6.3 Air Quality

7. How often have the following issues impacted your mental well-being?

Always - Often - Sometimes - Rarely - Never

7.1 Noise

7.2 Odors

7.3 Air Quality

8. Do you have a home garden where you grow food for your household to eat?

Yes

No

8. a) Do you use raised beds in your home garden?

Yes

No

8. b) What contaminants, if any, have you had your soil tested for? (If you have not had your soil tested, please enter "none".)

_____________________________________

9. Do you forage for plants or mushrooms to eat in your municipality?

Yes

No

10. How safe do you think produce grown in the soil of your municipality is to eat?

Very safe

Somewhat safe

Somewhat unsafe

Very unsafe

Unsure

11. Do you fish in the waterways of your municipality?

Yes

No

11. a) Do you eat the fish you catch in your municipality?

Yes

No

12. How safe do you think eating fish from your local waterways is?

Very safe

Somewhat safe

Somewhat unsafe

Very unsafe

Unsure

13. Do you swim or play in the waterways of your municipality?

Yes

No

14. How safe do you think swimming or playing in your local waterways is?

Very safe

Somewhat safe

Somewhat unsafe

Very unsafe

Unsure

15. How much do you trust the industrial facilities in your community to adhere to best practices or pollution standards?

Strong trust

Some trust

Neutral

Some mistrust

Strong mistrust

Unsure

Prefer not to say

16. How much do you trust the Pennsylvania Department of Environmental Protection to oversee the facilities in your community and hold them accountable?

Strong trust

Some trust

Neutral

Some mistrust

Strong mistrust

Unsure

Prefer not to say

17. How satisfied are you with the overall efforts of the government to protect the environment in your municipality?

Very satisfied

Somewhat satisfied

Neutral

Somewhat unsatisfied

Very unsatisfied

Unsure

18. Is there anything else you would like us to know about pollution/harmful chemicals in your community?

_______________________________________

**Your Current Life Situation**

1. Are you currently covered by any of the following types of health insurance or health coverage plans? Please select all that apply.^[[8]](#footnote-8)^

No, I do not currently have health insurance or a health coverage plan

Yes, insurance through a current or former employer or union (of yourself or another family member)

Yes, insurance purchased directly from an insurance company (by yourself or another family member

Medicare, for people 65 and older, or people with certain disabilities

Medicaid, Medical Assistance, or any kind of government-assistance plan for those with low incomes or a disability

TRICARE or other military health care

VA (enrolled for VA health care)

Other

Unsure

1. a) Which of the following reasons best describes the reason you do not currently have health insurance?^[[9]](#footnote-9)^

Another family member has health insurance, but it does not cover you

You can’t get coverage or were refused insurance due to poor health, illness, or age

It is too expensive

You don’t think you need it

You don’t know how to get insurance

You’re unemployed

Other

Unsure

2. Within the past 3 years, have you ever been denied insurance coverage for recommended medical treatment or medications?

Yes

No

Not applicable; I haven't had any recommended medical treatment in the past 3 years

Prefer not to say

3. Within the past 3 years, have you had to wait 3 or more months for a medical appointment?

Yes

No

Not applicable; I haven't tried to schedule any medical appointments in the past 3 years

Prefer not to say

4. Within the past 3 years, have you refused recommended medical treatment or medications as a result of the cost?^[[10]](#footnote-10)^

Yes

No

Not applicable; I haven't had any recommended medical treatment or medications in the past 3 years

Prefer not to say

5. What is your living situation today? Please select all that apply.

I own my own home

I am at risk of losing the home that I own

I am living in Section 8

I am living in public housing

I am renting my home

I am at risk of being evicted from the home I rent

I am having trouble affording the cost of my home

I do not have a steady place to live (I am temporarily staying with others, in a hotel, in a shelter, living outside on the street, on a beach, in a car, abandoned building, bus or train station, or in a park)

Prefer not to say

6. Think about the place you live. Do you have problems with any of the following? Please select all that apply. If other, please specify.^[[11]](#footnote-11)^

Bugs (e.g. roaches) or rodents

General cleanliness

Landlord disputes

Lead paint or pipes

Unreliable utilities (e.g. electricity, gas, heat)

Unaffordable utilities (e.g. electricity, gas, heat)

Medical condition that makes it difficult to live in current house

Mold

Oven or stove not working

Overcrowding

No or not working smoke detectors

Need for minor repairs

Need for major repairs

Water leak

Other _______________

Prefer not to say

No problems with the place I live

7. Within the past 12 months, you worried that your food would run out before you got money to buy more.^[[12]](#footnote-12)^

Never true

Sometimes true

Often true

Prefer not to say

8. Within the past 12 months, the food you bought just didn’t last and you didn’t have money to get more.^[[13]](#footnote-13)^

Never true

Sometimes true

Often true

Prefer not to say

9. In the past 12 months, has lack of transportation kept you from medical appointments, meetings, work or from getting things needed for daily living? Please select all that apply.^[[14]](#footnote-14)^

Yes, it has kept me from medical appointments or getting medications

Yes, it has kept me from non-medical meetings, appointments, work, or getting things that I need

No

10. What is your employment status today? Please select all that apply.

Employed full-time

Employed part-time

Self-employed

Out of work and looking for work

Out of work but not currently looking for work

Homemaker

Student

Retired

Military

Unable to work

11. How well does each of the following statements describe you or your situation?^[[15]](#footnote-15)^

Completely - Very well - Somewhat - Very little - Not at all – Prefer not to say

11.1 Because of my money situation, I feel like I will never have the things I want in life

11.2 I am just getting by financially

11.3 I am concerned that the money I have or will save won’t last

12. How often does this statement apply to you?^[[16]](#footnote-16)^

Always - Often - Sometimes - Rarely - Never

12.1 I have money left over at the end of the month

12.2 My finances control my life

13. While living in Southern Delaware County, have you ever been unable to receive the education or career skills training you wanted as a result of the cost or other barriers?

Yes

No

Not applicable; I have not wanted to receive any education or career skills training

14. While living in Southern Delaware County, how often have you experienced racism and/or discrimination?

Never

Rarely

Sometimes

Most of the time

Always

15. Have you been the victim of a violent crime while living in Southern Delaware County?

Yes

No

Prefer not to say

16. Which **three** of these factors do you think **most** **negatively** affect your health? Please select 3. If other, please specify.

Lack of reliable transportation

Limited educational opportunities

Limited financial resources

Limited job opportunities

Pollution and chemical exposures

Poor neighborhood conditions (e.g., trash, lack of

green space)

Poor quality housing

Problems accessing medical health care

Problems accessing mental health care

Problems with police or the criminal justice system

Systemic racism

Violence and/or crime

Other ______

**Community Priorities and Needs^[[17]](#footnote-17)^**

In this section, we would like you to think about the broader community in your municipality.

1. How true do you think each statement about health care is in your municipality?

Very true – Somewhat true – Neutral – Somewhat Untrue – Very Untrue

1.1 People have access to basic health care services, including dental, and preventative health care and screening.

1.2 People have access to specialized care services, such as allergists or orthopedists.

1.3 Mental health conditions are recognized and are **not** stigmatized in your municipality.

1.4 Mental health services are accessible to everyone in your municipality.

2. How true do you think each statement about health care is in your municipality?

Very true – Somewhat true – Neutral – Somewhat Untrue – Very Untrue

2.1 Safe housing (e.g., structurally sound, free from mold, well-ventilated) is available and accessible to everyone in our community.

2.2 Affordable housing is available and accessible to everyone in our community.

2.3 People of all ages and abilities have access to reliable public transportation.

2.4 People of all ages and abilities have access to safe public transportation.

3. How true do you think each statement about food and the environment is in your municipality?

Very true – Somewhat true – Neutral – Somewhat Untrue – Very Untrue

3.1 Healthy foods are affordable for all.

3.2 People in your municipality live within a mile of a grocery store that carries fresh produce.

3.3 Your municipality faces less or similar risk from pollution and chemical exposures as other communities.

3.4 Your municipality is acting to protect people from climate change.

3.5 People in your municipality live within a mile of a dedicated green space (e.g., park, garden).

3.6 Your municipality is clean.

3.7 The infrastructure (for example, sidewalks, lighting, trails) in your municipality makes it easy to be physically active.

4. How true do you think each statement about economic and educational opportunities is in your municipality?

Very true – Somewhat true – Neutral – Somewhat Untrue – Very Untrue

4.1 People are able to easily acquire full-time employment in your municipality.

4.2 People are able to earn $15 per hour or more in your municipality.

4.3 People have opportunities to build generational wealth in your municipality.

4.4 People have access to programs in your municipality that provide career skills training.

4.5 Quality education is available for all.

5. How true do you think each statement about economic and educational opportunities is in your municipality?

5.1 People feel safe in your municipality.

5.2 The infrastructure (for example, sidewalks, lighting, trails) in your municipality makes it safe and accessible for everyone to navigate.

5.3 There is **not** a lot of crime in your municipality.

5.4 Your municipality is free from graffiti or vandalism.

6. Over the past 5 years, do you think instances of racism have become more or less frequent in your municipality?

More frequent

Stayed the same

Less frequent

Unsure

I have not lived in Southern Delaware County for the past 5 years.

7. How serious do you think the problem of systemic racism in your municipality is?

Not at all serious

Somewhat not serious

Neutral

Somewhat serious

Very serious

Unsure

Not applicable; there is no systemic racism

8. Which **three** of these factors do you think policymakers in your community should prioritize? Please select 3.

Access to medical health care

Access to mental health care

Food insecurity

Housing instability

Lack of public transportation

Limited educational opportunities

Limited job opportunities

Minimum wage increase

Pollution and chemical exposures

Poor neighborhood conditions (e.g., trash, lack of

green space)

Poor quality housing

Police and/or criminal justice reform

Systemic racism

Violence and/or crime

Other

**Health Information**

- - - 1. How would you rate your overall health?^[[18]](#footnote-18)^

Excellent

Very Good

Good

Fair

Poor

Unsure

- - - 1. Over the **past 2 weeks**, how often have you been bothered by the following problems?^[[19]](#footnote-19)^

Not at all – Several days – More than half the days – Nearly every day

2.1 Feeling nervous, anxious or on edge

2.2 Not being able to stop or control worrying

2.3 Little interest or pleasure in doing things

2.4 Feeling down, depressed, or hopeless

- - - 1. While living in southern Delaware County, have you ever been diagnosed by a doctor or health professional with any of the following conditions? Please select all that apply.^[[20]](#footnote-20)^

Asthma

Allergies

Cancer

Chronic Obstructive Pulmonary Disease (COPD)

Diabetes

Heart Disease

Hypertension

Long COVID

Mental Health Condition (e.g., Depression, Anxiety)

Skin Disease (e.g., Dermatitis, Eczema, Rashes)

Other

3. a) What type(s) of cancer have you been diagnosed with?

_______________________________

3. b) If you feel comfortable sharing, what mental health condition(s) have you been diagnosed with?

_______________________________

3. c) What type(s) of skin disease have you been diagnosed with? _______________________________

3. d) What other condition(s) have you been diagnosed with?

_______________________________

- - - 1. Have you experienced pregnancy loss or complications during pregnancy while living in Southern Delaware County?

Yes

No

Prefer not to say

4. a) If you feel comfortable doing so, please describe your experience.

______________________________

5. Over the **past 4 weeks**, how often have you experienced the following symptoms?

Not at all Several days More than half the days Nearly every day

5.1 Brain fog

5.2 Coughing or wheezing

5.3 Dizziness

5.4 Eye irritation

5.5 Headache

5.6 Migraine

5.7 Nasal congestion (stuffy nose)

5.8 Nosebleed

5.9 Shortness of breath

5.10 Skin rash or itchiness

5.11 Sore throat

6. As far as you know, how many times have you had a COVID-19 infection?

______________________________

6. a) Do you believe you are experiencing effects of long COVID?

Yes

No

Unsure

6. b) Please describe the symptoms you associate with long COVID.

______________________________

7. During the **past 4 weeks**, to what extent has your physical health or emotional problems interfered with your normal social activities with family, friends, neighbors, or groups?^[[21]](#footnote-21)^

Not at all

Slightly

Moderately

Quite a bit

Extremely

8. During the **past 4 weeks**, have you had any of the following problems with your work or other regular daily activities **as a result of your physical health**?^[[22]](#footnote-22)^

Yes No

8.1 Cut down the **amount of time** you spent on work

**Accomplished less** than you would like

Were limited in the **kind** of work or other activities

Had **difficulty** performing the work or other activities (for example, it took extra effort)

9. During the **past 4 weeks**, have you had any of the following problems with your work or other regular daily activities **as a result of any emotional problems** (such as feeling depressed or anxious)?^[[23]](#footnote-23)^

Yes No

9.1 Cut down the **amount of time** you spent on work

9.2 **Accomplished less** than you would like

9.3 Didn’t do work or other activities as **carefully** as usual

**Child Health Information**

As far as you know, are you the only adult filling this out on behalf of the children in your home?

Yes

No

Unsure

Note: Participants who answered yes were not able to provide further information on their child(ren).

How many children (aged 0 to 17 years old) live in your household? __________________________________

(Please enter a whole number. If none, please enter 0.)

Note: Questions 2 to 11 were repeated for each reported child.

2. How old is your child? ___________

3. While living in southern Delaware County, has your child ever been diagnosed by a doctor or health professional with any of the following conditions? Please select all that apply.

Asthma

Allergies

Bronchitis

Cancer

Diabetes

Heart Disease

Hypertension

Long COVID

Mental Health Condition (e.g., Depression, Anxiety)

Reactive Airway Disease

Skin Disease (e.g., Dermatitis, Eczema, Rashes)

Other

No diagnoses while living in Southern Delaware County

3. a) What type(s) of cancer has your child been diagnosed with? ______________________

3. b) If you feel comfortable sharing, what mental health condition(s) has your child been diagnosed with?

______________________

3. c) What type(s) of cancer has your child been diagnosed with? ______________________

3. d) What other condition(s) has your child been diagnosed with? ______________________

4. In the past 12 months, how often has your child:^[[24]](#footnote-24),^^[[25]](#footnote-25)^

Never 1-2 times per year 3-12 times per year

More than 1 time per month More than 2 times per week Every day

4.1 Had wheezing (whistling sound from the chest) with a cold

4.2 Had wheezing (whistling sound from the chest) without a cold

4.3 Had an attack of wheezing that made it hard to breathe or catch his or her breath

4.4 Had a cough that would not go away

4.5 Complained that his or her chest felt tight or heavy

4.6 Wheezed with exercise or running or playing hard

4.7 Coughed with exercise or running or playing hard

4.8 Missed school due to asthma or other breathing issues

5. In the past 12 months, how often has your child’s sleep been disturbed because of wheezing, coughing, chest tightness, or shortness of breath?^[[26]](#footnote-26),^^[[27]](#footnote-27)^

Never

1-2 times per year

3-12 times per year

More than 1 time per month

More than 1 time per week

Every night

6. In the past 12 months, has your child taken any medication for asthma or other breathing issues?^[[28]](#footnote-28),^^[[29]](#footnote-29)^

Yes

No

7. Does your child currently use medications for asthma or other breathing issues on a daily basis?^[[30]](#footnote-30),^^[[31]](#footnote-31)^

Yes

No

8. In the past 12 months, how often have you missed work or other obligations due to your child’s asthma or other breathing issues?

Never

1-2 times per year

3-12 times per year

More than 1 time per month

More than 1 time per week

Every day

9. Over the **past 4 weeks**, how often has your child experienced the following symptoms?

Not at all Several days More than half the days Nearly every day

9.1 Brain fog

9.2 Dizziness

9.3 Eye irritation

9.4 Headache

9.5 Migraine

9.6 Nasal congestion (stuffy nose)

9.7 Nosebleed

9.8 Skin rash or itchiness

9.9 ` Sore throat

10. Does your child attend school where there are high rates of traffic/on a busy road?

Yes

No

Unsure

11. Does your child have adequate access to clean and safe drinking water both at home and at school?

Yes

No

Unsure

For the remaining questions in this section, please answer by considering all of your children.

12. With regard to pollution in the environment, how comfortable do you feel with your child/children playing outside in your municipality?

Very comfortable

Somewhat comfortable

Unsure

Somewhat uncomfortable

Very uncomfortable

Unsure

13. Is there a safe and accessible green space (e.g., a park) for your child/children to play within your municipality?

Yes

No

Unsure

14. Is there anything else you would like us to know about factors that affect your children’s health?

________________________

1. U.S. Census Bureau. 2020 Census Questionnaire. <<https://www2.census.gov/programs-surveys/decennial/2020/technical-documentation/questionnaires-and-instructions/questionnaires/2020-informational-questionnaire-english_DI-Q1.pdf>> (Last accessed on August 24, 2024). [↑](#footnote-ref-1)
2. U.S. Census Bureau, 2020 Questionnaire [↑](#footnote-ref-2)
3. Adapted from the National Health and Nutrition Examination Survey. 2017-March 2020 Data Documentation, Codebook, and Frequencies. <<https://wwwn.cdc.gov/Nchs/Nhanes/2017-2018/P_SMQ.htm#SMQ020>> (Last accessed on August 24, 2024). [↑](#footnote-ref-3)
4. Adapted from the National Health and Nutrition Examination Survey. 2017-March 2020 Data Documentation, Codebook, and Frequencies. [↑](#footnote-ref-4)
5. Adapted from the Center for Health, Environment and Justice (CHEJ). Unequal Response Unequal Protection: Investigating Health Impacts in Communities Exposed to Toxic Chemicals from Environmental Contaminants. <<https://drive.google.com/drive/folders/1PpQO8Ah8rwOTpP7UxCQr1dOOASjzVPdh?ths=true>> (Last accessed August 24, 2024). [↑](#footnote-ref-5)
6. CHEJ, Unequal Response Unequal Protection [↑](#footnote-ref-6)
7. Adapted from CHEJ, Unequal Response Unequal Protection [↑](#footnote-ref-7)
8. U.S. Census Bureau. The American Community Survey 2023 <<https://www2.census.gov/programs-surveys/acs/methodology/questionnaires/2023/quest23.pdf>> (Last accessed on August 24, 2024). [↑](#footnote-ref-8)
9. Kaiser Family Foundation. Health Insurance Survey. <<https://www.kff.org/wp-content/uploads/2013/01/2003-health-insurance-survey-toplines.pdf>> (Last accessed August 24, 2024). [↑](#footnote-ref-9)
10. Adapted from Billioux, Alexander, Katherine Verlander, Susan Anthony, and Dawn Alley. "Standardized screening for health-related social needs in clinical settings: the accountable health communities screening tool." *NAM Perspectives* (2017). [↑](#footnote-ref-10)
11. Adapted from Billioux, “Standardized screening for health-related social needs” [↑](#footnote-ref-11)
12. Hager, Erin R., Anna M. Quigg, Maureen M. Black, Sharon M. Coleman, Timothy Heeren, Ruth Rose-Jacobs, John T. Cook et al. "Development and validity of a 2-item screen to identify families at risk for food insecurity." *Pediatrics* 126, no. 1 (2010): e26-e32. [↑](#footnote-ref-12)
13. Hager, “Development and validity of a 2-item screen” [↑](#footnote-ref-13)
14. Billioux, Alexander, Katherine Verlander, Susan Anthony, and Dawn Alley. "Standardized screening for health-related social needs in clinical settings: the accountable health communities screening tool." *NAM Perspectives* (2017). [↑](#footnote-ref-14)
15. Consumer Financial Protection Bureau. Measuring financial well-being: A guide to using the CFPB Financial Well-Being Scale 2015 <<https://files.consumerfinance.gov/f/201512_cfpb_financial-well-being-user-guide-scale.pdf>> (Last accessed on August 24, 2024). [↑](#footnote-ref-15)
16. Consumer Financial Protection Bureau, “Measuring financial well-being” [↑](#footnote-ref-16)
17. Approach in this section was derived from University of Kansas Work Group on Health Promotion and Community Development. Neighborhood Concerns Index 1997 <<https://ctb.ku.edu/sites/default/files/chapter_files/neighborhood_concerns_index.pdf>> (Last accessed on August 24, 2024). [↑](#footnote-ref-17)
18. RAND Corporation. 36-Item Short Form Survey Instrument (SF-36). RAND; 2017. [↑](#footnote-ref-18)
19. Kroenke, Kurt, Robert L. Spitzer, Janet BW Williams, and Bernd Löwe. "An ultra-brief screening scale for anxiety and depression: the PHQ–4." *Psychosomatics* 50, no. 6 (2009): 613-621. [↑](#footnote-ref-19)
20. Adapted from Resnick, Beth, Patti Truant, Jennifer Le, and Mary Fox. Follow-up on Spring Valley Health Study. <<https://doee.dc.gov/sites/default/files/dc/sites/ddoe/publication/attachments/JHU%20Community%20Survey%20Report%20FINAL.pdf>> (Last accessed August 24, 2024). [↑](#footnote-ref-20)
21. RAND Corporation. 36-Item Short Form Survey Instrument (SF-36). RAND; 2017. [↑](#footnote-ref-21)
22. RAND, SF-36 [↑](#footnote-ref-22)
23. RAND, SF-36 [↑](#footnote-ref-23)
24. Lewis, Toby C., Thomas G. Robins, Christine LM Joseph, Edith A. Parker, Barbara A. Israel, Zachary Rowe, Katherine K. Edgren, Maria A. Salinas, Michael E. Martinez, and Randall W. Brown. "Identification of gaps in the diagnosis and treatment of childhood asthma using a community-based participatory research approach." *Journal of Urban Health* 81 (2004): 472-488. [↑](#footnote-ref-24)
25. National Asthma Education and Prevention Program. "Guideline for the Diagnosis and Management of Asthma." *Expert Panel Report 3* (2007). [↑](#footnote-ref-25)
26. Lewis, “Identification of gaps in the diagnosis” [↑](#footnote-ref-26)
27. National Asthma Education and Prevention Program. "Guideline for the Diagnosis and Management of Asthma."  [↑](#footnote-ref-27)
28. Lewis, “Identification of gaps in the diagnosis” [↑](#footnote-ref-28)
29. National Asthma Education and Prevention Program. "Guideline for the Diagnosis and Management of Asthma."  [↑](#footnote-ref-29)
30. Lewis, “Identification of gaps in the diagnosis” [↑](#footnote-ref-30)
31. National Asthma Education and Prevention Program. "Guideline for the Diagnosis and Management of Asthma."  [↑](#footnote-ref-31)
